# Current animal models of hemophilia: the state of the art

**DOI:** 10.1186/s12959-016-0106-0

**Published:** 2016-10-04

**Authors:** Ching-Tzu Yen, Meng-Ni Fan, Yung-Li Yang, Sheng-Chieh Chou, I-Shing Yu, Shu-Wha Lin

**Affiliations:** 1 Department of Clinical Laboratory Science and Medical Biotechnology, College of Medicine, National Taiwan University, Taipei, Taiwan; 2 Center of Genomic Medicine, National Taiwan University, Taipei, Taiwan; 3 Department of Laboratory Medicine, National Taiwan University Hospital, College of Medicine, National Taiwan University, Taipei, Taiwan; 4 Department of Pediatrics, National Taiwan University Hospital, College of Medicine, National Taiwan University, Taipei, Taiwan; 5 Department of Internal Medicine, National Taiwan University Hospital, College of Medicine, National Taiwan University, Taipei, Taiwan; 6 Laboratory Animal Center, College of Medicine, National Taiwan University, Taipei, Taiwan

**Keywords:** Hemophilia, Animal models, Genetically-engineered, CRISPR/Cas9

## Abstract

Hemophilia is the most well-known hereditary bleeding disorder, with an incidence of one in every 5000 to 30,000 males worldwide. The disease is treated by infusion of protein products on demand and as prophylaxis. Although these therapies have been very successful, some challenging and unresolved tasks remain, such as reducing bleeding rates, presence of target joints and/or established joint damage, eliminating the development of inhibitors, and increasing the success rate of immune-tolerance induction (ITI). Many preclinical trials are carried out on animal models for hemophilia generated by the hemophilia research community, which in turn enable prospective clinical trials aiming to tackle these challenges. Suitable animal models are needed for greater advances in treating hemophilia, such as the development of better models for evaluation of the efficacy and safety of long-acting products, more powerful gene therapy vectors than are currently available, and successful ITI strategies. Mice, dogs, and pigs are the most commonly used animal models for hemophilia. With the advent of the nuclease method for genome editing, namely the CRISPR/Cas9 system, it is now possible to create animal models for hemophilia other than mice in a short period of time. This review presents currently available animal models for hemophilia, and discusses the importance of animal models for the development of better treatment options for hemophilia.

## Background

Hemophilia, caused by deficiency or dysfunction of coagulation factor VIII (FVIII) or factor IX (FIX), is the most well-known hereditary bleeding disorder, with an incidence of one in every 5000 to 30,000 males worldwide. Current standard care involves intravenous infusions of protein products on demand, and prophylaxis. Although very successful, protein replacement therapy runs the risk of inducing neutralizing antibodies or “inhibitors”. Patients with inhibitors can no longer use FVIII/FIX concentrates and require more expensive bypass products [1] or elimination of the inhibitors by immune-tolerance induction (ITI) [2]. ITI may not be performed due to economical accessibility (affordability) of FVIII/FIX products and, in addition, ITI of hemophilia-B is performed less often because inhibitors are less well-developed and have lower success rates. Multiple factors are involved in the success of ITI of hemophilia A including the historical peak and pre-ITI level of inhibitors, age of patients, time period from inhibitor diagnosis to ITI, interruptions of ITI and peak inhibitors during ITI. Products and regimen may also play a role in ITI outcome, although current clinical data are contradictory or inconclusive with regard to this point.

Considerable effort has been exerted to improve hemophilia treatment through the development of long-acting products, powerful gene therapy vectors, and successful ITI strategies. Clinical trials aiming to tackle these challenges have been undertaken, and many preclinical trials have been carried out on animal models for hemophilia generated by the research community. Preclinical and clinical trials all require a suitable animal model of hemophilia. The most commonly used animal models for hemophilia include but are not limited to mice, dogs, and pigs. Animal models are important for basic biological and preclinical studies, such as studies aimed at understanding the pathophysiology of inhibitor formation, improving ITI protocols, estimating drug doses, and evaluating therapeutic efficacy before human trials. With the advent of the nuclease method for genome editing, named the CRISPR/Cas9 system, it is now possible to create animal models of hemophilia other than mice in a short period of time. This review presents an overview of currently available animal models for hemophilia, and discusses the importance of animal models for the development of better treatment options for hemophilia.

## Review

### Hemophilia animals with spontaneous mutations

#### Hemophilia A dogs

Dogs with naturally occurring hemophilia A were first documented following observations of abnormally prolonged bleeding that could be treated or prevented by infusion of canine FVIII. Currently, two colonies of hemophilia A dogs are most commonly used in studies: an Irish Setter colony maintained at the University of North Carolina in Chapel Hill [3], and a miniature schnauzer colony maintained at Queens University in Toronto, Canada [4]. Both colonies are deficient in circulating FVIII and show an aberrant inversion mutation between ~0.5 Mb upstream of the *FVIII* gene and its intron 22 [5, 6]. In addition to intron 22 inversion of *FVIII* gene, dogs with hemophilia A have also been described to have other spontaneous mutations in the *FVIII* gene [7, 8].

Hemophilia A dogs have been used extensively in preclinical trials of human FVIII protein products as well as in studies on the safety and efficacy of adeno-associated viral (AAV) vector-conducted gene therapy [9], in testing the gene therapy of platelet-specific expression of human FVIII [10], and have provided promising data for bypass therapy [11]. A subset of these dogs have a propensity to develop inhibitors [12] after infusion of canine FVIII [13, 14], with the Queens colony being more prone to inhibitor development than the Chapel Hill dogs. Dogs with inhibitors can facilitate the studies of inhibitor pathogenesis and ITI strategies, e.g., eradication of pre-existing neutralizing antibodies by liver gene therapy [15]. The development of inhibitors to FVIII is the most significant complication of protein replacement therapy. Because of the high homology of the canine and human immune system, assessments of FVIII immunogenicity using hemophilia A dogs may provide meaningful insights into the human immune response.

#### Hemophilia A sheep

Phenotypes consistent with human hemophilia A, including spontaneous bleeding and plasma FVIII activity of less than 1 %, have been found in sheep [16, 17]. Sheep with naturally occurring hemophilia A were re-established through reproductive technologies in 2009. Sequence mapping revealed a single nucleotide insertion-induced frame shift of the *FVIII* gene creating a premature stop codon at the base position 3112–4 in exon 14 and five additional stop codons within the next 183 bp [18]. Hemophilia sheep have been used in studies relevant to gene and cell therapies for hemophilia, including investigations of pre-existing immunity to AAV vectors [19] and the use of mesenchymal stem cells as cellular delivery vehicles for the *FVIII* gene [20]. However, the restricted availability of the recombinant ovine FVIII for treatment may limit the practical use of hemophiliac sheep.

#### Hemophilia A rats

Researchers identified an inbred WAG/RijY rat strain, designated WAG/RijYcb, which tended to show abnormal hemorrhaging, a prolonged activated partial thromboplastin time, and a mutated *FVIII* gene (proline was substituted for leucine at amino acid 176 in the A1 domain). In rats, the *FVIII* gene is located on chromosome 18. Thus, hemophilia in rats is autosomal recessive, which contrasts with humans and other animal models where it is an X-linked hereditary disease [21, 22]. The mutated FVIII region of WAG/RijYcb rats generally lacks immunodominant epitopes for inhibitor formation thus this model may not be suitable for studying the immune response to FVIII treatment or developing ITI strategies.

#### Hemophilia B dogs

Similar to human hemophilia B, canine hemophilia B has a sex-linked inheritance pattern, and no detectable circulating FIX exists in the plasma. Researchers have identified at least three colonies of hemophilia B dogs, each of which has a unique molecular FIX defect. Hemophilia B cairn terriers were identified in Toronto [23] and have been maintained in Chapel Hill since 1966 [24]. These animals have a point mutation that results in substitution of glutamic acid for glycine at AA 377 in the catalytic domain [24]. Lhasa Apso dogs with hemophilia B, established in Auburn, Alabama [25], carry a 5-bp deletion at nucleotides 772–776 and a C-to-T transition in the *FIX* gene, resulting in a premature stop codon at AA 146 [25]. A Labrador retriever colony at Cornell University, New York [26], carries complete deletion of the canine *FIX* gene. Chapel Hill and Auburn dogs have been used extensively for testing FIX products and gene therapy strategies. The Auburn dogs are prone to developing inhibitors to infused canine FIX [27, 28]. Translational data produced from hemophilia B dogs have supported the development of long-acting FIX [29, 30] with accompanying recent human clinical trials [12, 31–34]. In contrast to the higher success ITI rate in hemophilia A, the ITI outcome in hemophilia B patients with FIX inhibitors is poor and research in this area remains in its infancy [35]. Recently, an ITI strategy using AAV liver expression of a FIX variant, FIX-Padua was successfully applied to hemophilia B dogs prone to inhibitors. This strategy eradicated FIX inhibitor in dogs with pre-existing inhibitors [34], suggesting its value in the development of ITI.

### Genetically engineered animal models of hemophilia

#### Hemophilia A mice

As small mammals that are easy to breed and relatively inexpensive to maintain in large numbers, mice are a popular animal for medical research and preclinical testing worldwide. Although spontaneous bleeding does not naturally occur in mice, genome-editing technologies have contributed to development of various hemophiliac mouse models. Hemophilia mice are the best initial model to use when attempting to test new therapeutics because they only require small amounts of drugs.

In 1995, the first two hemophilia A mouse models, with a mixed genetic background of 129SV and C57BL/6, were established. These two strains of FVIII knockout mice were generated through gene targeting and embryonic stem cell manipulation [36]. A neo cassette was inserted into the 3′ end of exon 16 of the mouse *FVIII* gene in one strain (E16 mice), whereas exon 17 was disrupted in the other strain (E17 mice). Mice had no detectable circulating FVIII, and their plasma FVIII activity was less than 1 %. Unlike human hemophilia A, little spontaneous bleeding is observed in mouse models of hemophilia A, whereas tail clipping or other invasive procedures could lead to death. Mouse models of hemophilia A are widely used for the evaluation of FVIII treatment efficacy [37, 38], investigation of mechanisms of inhibitor formation, and development of ITI protocols for FVIII [39].

To mimic patients with severe hemophilia A who express no endogenous FVIII (i.e., cross-reacting material) and are prone to form inhibitors after protein replacement therapy, researchers generated a total *FVIII* gene knockout hemophilia A mouse model in a pure C57BL/6 background. The entire coding sequence was deleted by a Cre recombinase-LoxP site-mediated deletion. Plasma FVIII activity and anti-FVIII inhibitor titer induced after FVIII treatment were comparable to those of E16 mice [40].

#### Hemophilia A pig

Pigs with hemophilia A were generated by nuclear transfer and cloning from porcine fetal fibroblasts carrying disruption of exon 16 of the porcine *FVIII* gene by the neomycin-resistant gene [41]. Species differences between humans and mice, such as size, general physiology, anatomy, and lifespan, limit the value of mouse models in preclinical trials. In contrast, the coagulation systems of pigs and humans are highly homologous. Moreover, hemophilia A pigs may develop arthropathy similar to humans because of repeated joint bleeding [41]. Thus, hemophilia A pigs provide another option for evaluating novel therapeutics for hemophilia A patients [42].

#### Hemophilia B mice

Although mice with naturally occurring deficiency of FIX have not been identified, a series of FIX knockout mice were engineered by homologous recombination in embryonic stem cells. Initially, the mouse *FIX* gene was disrupted by insertion of a neo cassette into exon 3 [43] or the coding region for the catalytic domain of FIX [44, 45], resulting in a mouse model with no detectable mRNA or plasma protein expression of FIX. In addition to mice with complete deletion of FIX, researchers have created knock-in mice expressing human FIX carrying a missense mutation (R333Q-hFIX) under the control of the mouse FIX promoter. The R333Q mutation is located in the catalytic domain of human FIX; the same mutation has been identified in several patients with severe hemophilia B. In R333Q hFIX mice, mutant human FIX transcript and circulating human FIX protein were detectable throughout development, but the FIX protein activity was <1 % [46]. Similar to hemophilia A mice, hemophilia B mice do not show spontaneous bleeding, but will bleed and die after tail clipping unless the wound is cauterized. Hemophilia B mice have been used to test the efficacy of FIX and FIX variants, including those FIX variants with very high clotting activities [47–52], as well as to evaluate the immunity and safety of gene therapy [53–55].

Researchers have also established several knock-in mice carrying a FIX variant coding for K5A in the Gla domain of FIX [56], a full-length wild-type (WT) human FIX coding sequence, and a FIX variant expressing FIX-Triple containing 3 amino acid modifications [47]. The K5A mutation impairs FIX binding to collagen type IV, and these mice show a relatively mild bleeding phenotype [56]. The knock-in mice with complete human WT and FIX-Triple FIX coding sequence exhibited clotting activity of, respectively, nearly 5 and 50 % that of mouse FIX, which was unexpected and was perhaps due to species specificity of FVIII [47].

#### Humanized hemophilia A mice for FVIII immunity

To understand the regulation of antibody responses against FVIII in hemophilia A, researchers modified the hemophilia A mouse model to be “humanized” for HLA class II antigen. Researchers crossed E17 mice with mice expressing chimeric human-mouse HLA-DRB1*1501, which is associated with an increased risk of inhibitor development in humans [57]. Use of this humanized hemophilia A mouse model allowed identification of immunodominant FVIII peptides that trigger inhibitor formation, as well as the characterization of interactions of T-cell receptors with disease-associated FVIII peptides and MHC class II molecules [58].

### Novel hemophilia NSG mouse models established by CRISPR/Cas9 technology

**Fig. 1:**
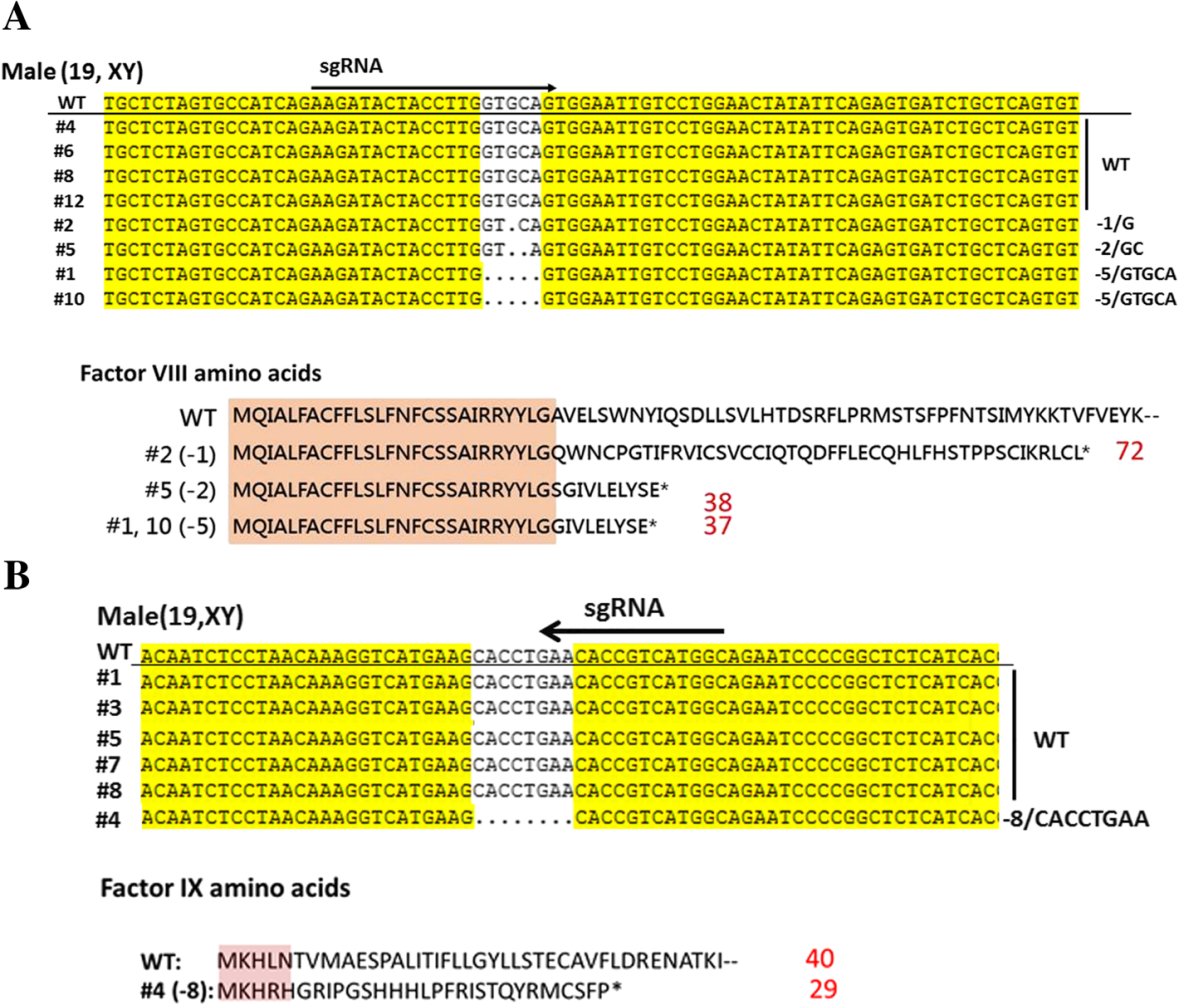
Illustration of NSG male mouse with missense mutation in exon 1 of the mouse *FVIII* or *FIX* gene. Sequences of tail DNA derived from male founders of hemophilia NSG mice are shown. **a** Four male founders carrying mutations in the *FVIII* gene were generated. Deletion of 1 bp (−1/G), 2 bp (−2/GC), and 5 bp (−5/GTGCA) in exon 1 created a premature stop codon at the 72, 38 and 37^th^ AA residue of mouse FVIII, respectively. **b** One male founder carried an −8/CACCTGAA deletion in the *FIX* gene, which resulted in a premature stop codon at the 30^th^ AA residue of mouse FIX

A recent advance in genetic engineering technology provides a powerful tool to modify the genome in any living species [59]. CRISPR (clustered regularly interspaced short palindromic repeat)-associated RNA-guided endonuclease Cas9, which was identified from the microbial adaptive immune system [60], can be used to alter the mammalian genome with high efficiency and precision. Recently, a reliable animal model for elucidating the humoral and cellular immune responses of patients to FVIII/FIX treatment was developed. The CRISPR/Cas9 system and immunodeficient NSG mice (Nod/Scid-Il2γ^−/−^) were combined to mutate the *FVIII* and *FIX* genes, generating hemophilia A/B mice with the NSG background (HemoA/B-NSG mice). Oligonucleotides of 20 residues serving as specific guiding RNAs (gRNAs) were developed to target exon 1 of mouse *FVIII* and *FIX*. The gRNA and CRISPR/Cas9 RNA were microinjected into the NSG mouse zygotes to generate founders. Four male founder mice were obtained, each carrying a 1, 2 or 5-bp deletion in exon 1 of the *FVIII* gene, resulting in a premature stop codon (Fig. 1a). Hepatic FVIII mRNA level of these mice was 50 % lower than that of NSG mice (unpublished data), whereas their plasma FVIII activity was dramatically decreased to be comparable to that of hemophilia A mice with the 129SV/C57BL/6J mixed genetic background [36]. Two male founder mice were identified that had the same mutation, i.e., carrying an 8-nucleotide deletion in exon 1 of the *FIX* gene, which created a premature stop codon (Fig. 1b). NSG mice are more acceptable than NOD/SCID mice for transplanting with human hematopoietic cells and can, therefore, be used for direct assessment of the human immune response to FVIII/FIX treatment.

## Conclusions

Translational research from hemophilia animal models gives valuable information about safety and efficacy, and guides design of human clinical trials. Despite their availability, hemophilia animal models have many potential disadvantages and limitations, including a short half-life of human FVIII (in mice), differences in tissue tropism of viral vectors compared to humans, and immune reactions to human FVIII/FIX. Reducing inhibitor incidence and elimination of inhibitors in hemophilia patients is a major task that needs to be resolved. It is considered that the use of different products and treatment protocols may be involved in inhibitor production [61], but the possible mechanism(s) is not fully understood. An appropriate humanized animal model for the evaluation of different products, regimens, and ITI strategies is still lacking. CRISPR/Cas9 technology is a very efficient method to generate hemophilia in rare and difficult-to-breed mice. We believe that our hemophilia NSG mice will be a very useful model for studying the human immune response to therapeutics.

## Abbreviations

FVIII: Factor VIII
FIX: Factor IX
CRISPR/Cas9: Clustered regularly interspaced short palindromic repeat-associated RNA-guided endonuclease Cas9
AA: Amino acid
ITI: Immune tolerance induction
